# Beneficial Effects of Simulated Gastro-Intestinal Digests of Fried Egg and Its Fractions on Blood Pressure, Plasma Lipids and Oxidative Stress in Spontaneously Hypertensive Rats

**DOI:** 10.1371/journal.pone.0115006

**Published:** 2014-12-11

**Authors:** Forough Jahandideh, Kaustav Majumder, Subhadeep Chakrabarti, Jude S. Morton, Sareh Panahi, Susan Kaufman, Sandra T. Davidge, Jianping Wu

**Affiliations:** 1 Department of Agricultural, Food and Nutritional Science, University of Alberta, Edmonton, Alberta, Canada; 2 Department of Obstetrics and Gynecology, University of Alberta, Edmonton, Alberta, Canada; 3 Department of Physiology, University of Alberta, Edmonton, Alberta, Canada; 4 Cardiovascular Research Centre, University of Alberta, Edmonton, Alberta, Canada; 5 Women & Children’s Health Research Institute, University of Alberta, Edmonton, Alberta, Canada; Max-Delbrück Center for Molecular Medicine (MDC), Germany

## Abstract

**Background:**

We have previously characterized several antihypertensive peptides in simulated digests of cooked eggs and showed blood pressure lowering property of fried whole egg digest. However, the long-term effects of this hydrolysate and its fractions on blood pressure are not known. Therefore, the objectives of the study were to determine the effects of long term administration of fried whole egg hydrolysate and its fractions (i.e. egg white and egg yolk) on regulation of blood pressure and associated factors in cardiovascular disease such as plasma lipid profile and tissue oxidative stress.

**Methods and Results:**

We used spontaneously hypertensive rats (SHR), an animal model of essential hypertension. Hydrolysates of fried egg and its fractions were prepared by simulated gastro-intestinal digestion with pepsin and pancreatin. 16–17 week old male SHRs were orally administered fried whole egg hydrolysate, non-hydrolyzed fried whole egg, egg white hydrolysate or egg yolk hydrolysates (either defatted, or not) daily for 18 days. Blood pressure (BP) and heart rate were monitored by telemetry. Animals were sacrificed at the end of the treatment for vascular function studies and evaluating plasma lipid profile and tissue oxidative stress. BP was reduced by feeding fried whole egg hydrolysate but not by the non-hydrolyzed product suggesting a critical role for *in vitro* digestion in releasing anti-hypertensive peptides. Egg white hydrolysate and defatted egg yolk hydrolysate (but not egg yolk hydrolysate) also had similar effects. Reduction in BP was accompanied by the restoration of nitric oxide (NO) dependent vasorelaxation and reduction of plasma angiotensin II. Fried whole egg hydrolysate also reduced plasma levels of triglyceride although it was increased by the non-hydrolyzed sample. Additionally the hydrolyzed preparations attenuated tissue oxidative stress.

**Conclusion:**

Our results demonstrate that fried egg hydrolysates exert anti-hypertensive effects, improve plasma lipid profile and attenuate tissue oxidative stress *in vivo*.

## Introduction

Hypertension, defined as the persistent elevation of systolic/diastolic blood pressure over 140/90 mmHg, is a major risk factor for developing cardiovascular diseases [1]. Although no definite cause is apparent in the majority of hypertension cases (i.e. idiopathic hypertension), a number of underlying pathologies such as hyperactivity of the renin angiotensin system (RAS), inflammation, oxidative stress and impaired vascular relaxation, can contribute to its onset and long-term persistence [2],[3]. Although various pharmaceutical drugs are available for treatment of hypertension, management of hypertension often requires lifelong adherence to the medication and is associated with significant adverse side-effects [4]. Thus, there has been a growing interest in using alternative options, such as adoption of a healthy lifestyle with restricted energy and dietary sodium intake, engagement in physical activities and stopping smoking, for prevention and management of hypertension [5],[6]. The antihypertensive properties of several food sources including grain, vegetables and fruits, dairy products, fish, meat, and egg, legumes, nuts, and seeds have been studied extensively (reviewed by [7],[8]). In a previous study, we have shown that upon comparison of popular methods for cooking eggs, frying rather than boiling was more effective in generating ACE-inhibitory peptides on subsequent enzymatic hydrolysis [9]. Our further study showed that the fried whole egg digest could lower blood pressure in spontaneously hypertensive rats (SHRs) in a 3-day trial [10]. However, the long-term effects of this digest on regulating blood pressure and other cardiovascular parameters remain unknown.

Blood pressure is regulated through several mechanisms including modification of ACE activity and vascular function as well as changes in oxidative status. Antioxidants may decrease blood pressure through decreasing oxidative stress in the body thus preserving the activity of nitric oxide synthase (NOS) as well as increasing nitric oxide (NO) bioavailability. Moreover, the potential interrelations between blood pressure and plasma lipids may contribute to increased coronary heart disease in hypertensive patients (Bonna and Thelle, 1991). Given this background, we aimed to determine the effects of long-term administration of fried whole egg hydrolysate on regulation of blood pressure and associated factors such as plasma lipid profile and tissue oxidative stress. Since whole egg contains egg white and egg yolk fractions with different constituents and functionality, we were also interested to verify the relative contributions of egg fractions in mediating these effects.

## Materials and Methods

### Sample preparation

Fresh white-shell chicken eggs were obtained from the Poultry Research Centre of the University of Alberta (Edmonton, AB, Canada). Whole eggs (homogenized and non-homogenized) were placed in a pre-heated frying pan (180°C) for 40 sec each side without adding oil; after cooling to room temperature, egg whites and yolks were separated from the non-homogenized samples and frozen immediately at −20°C until further use. To prepare defatted egg yolk samples, lipids were extracted from fried egg yolks using ethanol as described by Palacios et al. [11] with some modifications. Ethanol (95%) was added to fried egg yolks (30 g/100 mL) and stirred for one hour. The mixture was then centrifuged at 1900×g for 5 min. The supernatant containing lipids was removed and the precipitate was mixed with another volume of ethanol and repeated for three extractions. The precipitate was then dried under nitrogen stream (to evaporate out the ethanol), and stored at −20°C for further experiments.

### Simulated gastro-intestinal digestion

Each cooked egg sample (whole eggs, their egg whites and yolks) were digested under simulated gastro-intestinal conditions involving sequential treatments with pepsin (porcine gastric mucosa; Sigma-Aldrich, Oakville, ON, Canada) and pancreatin (porcine pancreas; Sigma-Aldrich, Oakville, ON, Canada) as described in our previous study [9]. The pH and temperature of the samples were maintained constantly during the course of hydrolysis using Titrando (Metrohm, Herisan, Switzerland) and a circulating water bath respectively. The hydrolysis was terminated by raising the temperature to 95°C and the hydrolysates were freeze-dried without centrifugation separation.

### Animal model and surgery

Twelve to fourteen week old male spontaneous hypertensive rats (SHRs, 290.0±10.5 g) were obtained from Charles River (Senneville, QC, Canada). After arrival, they were acclimatized for one week at the University of Alberta animal facility, exposed to a 12∶12 hour cycle of light:dark in a humidity and temperature-controlled (60% RH, and 23°C) environment. Rats were maintained on standard chow (0.3% NaCl) and water *ad libitum*. After one-week acclimation, these animals were chronically implanted with DSI telemetry transmitters (PA-C40; Data Sciences International, Minneapolis, MN) as we previously described [12] and then randomly assigned to treatment groups after a one week period of recovery following surgery. The first phase study was aimed to evaluate the long term *in vivo* efficacy of fried whole egg hydrolysate. SHR animals were assigned into 2 groups: control (n = 6), and fried whole egg hydrolysate (n = 7, FWE-H, 1000 mg/kg BW); this dose was chosen based on our previously 3-day study [10]. The 2^nd^ phase study was aimed to determine the effect of enzymatic digestion on the blood pressure lowering effect. In this study, animals were also assigned into two groups: control (n = 3), and non-hydrolyzed fried whole egg (n = 6, FWE-NH, 1000 mg/kg BW). The 3^rd^ phase study was aimed to determine the egg fractions (whites, yolks, and defatted yolk) responsible for the blood pressure lowering effects. In this study, animals were randomly assigned into four groups: control (n = 5), egg white hydrolysate (n = 6, EW-H), egg yolk hydrolysate (n = 6, EY-H), and defatted egg yolk hydrolysate (n = 7, DEY-H). As the three phases of the study were not performed at once, each phase had its own control group.

All treatments were given once per day at a dosage of 1000 mg/kg BW for 18 days after mixing with 20 mL of Ensure (Abbott Nutrition, QC, Canada). Control animals received Ensure only at the same volume. Blood pressure (BP) was recorded for 24 h (10 sec of every 1 min) on days 0 (baseline), 3, 6, 9, 12, 15, and 18 for all animals. On the morning of day 19, the animals were euthanized by exsanguination via excision of the heart under inhaled isoflurane anesthesia. Following sacrifice, blood was collected from the heart in ethylenediaminetetraacetic acid (EDTA) coated tubes (BD Vacutainer, NJ, USA), and centrifuged (1,000×g for 20 min at 4°C) to obtain plasma while tissues were removed immediately, rinsed with cold saline, weighed, flash frozen with liquid nitrogen and stored at −80°C for further analysis. The mesenteric arteries were also isolated and used immediately for *ex vivo* vascular function studies.

### Ethics statement

The experimental procedures were approved by the University of Alberta Animal Welfare Committee (Protocol # 611/09/10D) in accordance with the guidelines issued by the Canadian Council on Animal Care and also adhered to the Guide for the Care and Use of Laboratory Animals published by the United States National Institutes of Health.

### Data acquisition and signal processing

BP recording was performed in a quiet room with minimal electrical interference. Individual rat cages were placed on top of a receiver (Model RPC-1, ADI instruments, CO, USA) in order to record the signals for the measurement of various cardiovascular parameters through a pressure output adaptor (Model R11CPA, ADI instruments) as described previously [12]. Each telemetry device was calibrated and verified. An atmospheric-pressure monitor (Model APR-1, ADI instruments) was also installed to normalize the blood pressure values received from the transmitters. This normalization provides the actual BP values irrespective of changes in atmospheric pressure. The experimental data were recorded continuously in real time using the data acquisition software LabChart version 7.3 (ADI instruments). Systolic blood pressure (SBP), diastolic blood pressure (DBP) and mean arterial blood pressure (MAP) were obtained from the observed signal. The heart rate (HR) was calculated between two consecutive points and expressed in beats per minute (bpm).

### Vascular function studies

*Ex vivo* vascular reactivity experiments were carried out on second order branches of the mesenteric arteries. Following animal sacrifice, the proximal part of the small intestine and its associated vascular arcade was quickly removed and transferred into a silicone coated petri-plate containing ice-cold HEPES-buffered phosphate saline solution (HEPES-PSS) containing 142 mM NaCl, 4.7 mM KCl, 1.17 mM MgSO_4_, 4.7 mM CaCl_2_, 1.18 mM K_2_PO_4_, 10 mM HEPES and 5.5 mM glucose at pH 7.4. Small mesenteric arteries (internal diameter ranging from 150 to 250 µm) were carefully cleaned of all surrounding adipose and connective tissues, dissected into approximately 2 mm sections, mounted on two 40 µm tungsten wires (Fine Wire Company, California, USA) and attached to a wire-myograph (DMT, Copenhagen, Denmark) to allow isometric tension recordings. Vessels were normalized stepwise to determine their optimal resting tension, set to 0.8×IC100 (the internal circumference (IC) equivalent to 100 mm Hg). Mesenteric arteries were exposed to a single dose of phenylephrine (PE; 10 µmol/L; Sigma Aldrich, Oakville, Canada) twice, followed by a single dose of methacholine (MCh; 3 µmol/L; Sigma) following a 30 min equilibration, to assess the functional integrity of the endothelium and smooth muscle. Constrictor responses were determined by performing a cumulative concentration response curve to PE (10^−8^ to 10^−4 ^mol/L). The role of nitric oxide (NO) in endothelium-dependent relaxation was assessed via studying the MCh-relaxation response of vessels in the presence or absence of the nitric oxide synthase (NOS) inhibitor N-nitro-L-arginine methyl ester (L-NAME, 100 µmol/L, Sigma). After incubation of vessels with/without L-NAME, all vessels were pre-constricted using PE (80% of individual vessel’s maximum constriction) until they reached a plateaued response. Cumulative doses of MCh (10^−10^ to 10^−4 ^mol/L) were then added to the bath to assess the cumulative concentration response curve to MCh. At the end of the experiment, the vessels were exposed to high potassium buffer to confirm their viability. Vessels with constriction less than 80% of their maximum constriction were excluded from analysis.

### Plasma analysis

Following animal sacrifice, blood samples were collected and centrifuged (1,000×g for 20 min at 4°C) to obtain the plasma. Plasma was then stored at −80°C until further analysis. Angiotensin II (Ang II) was quantified by ELISA kits (Ang II ELISA, Cayman Chemical, Ann Arbor, MI, USA) based on the manufacturer’s instruction. Triglycerides (TG) concentration (WAKO Chemicals USA, Richman, VA, USA; Cat # 461-08992/461-09092) as well as total cholesterol (WAKO; Cat # 439-17501), low-density lipoprotein cholesterol (LDL-C) (WAKO; Cat # 993-00404/999-00504) and high-density lipoprotein cholesterol (HDL-C) (WAKO; Cat # 997-72591/993-72691) concentrations were all measured using direct colorimetric enzymatic reactions as per the manufacturer’s instructions.

### Markers of lipid peroxidation in tissues

Tissue (kidneys and heart) homogenates were prepared by sonication after addition of Radio-Immunoprecipitation Assay (RIPA) buffer (Sigma Aldrich, Oakville, Canada), in the presence of a protease inhibitor cocktail (Sigma Aldrich, Oakville, Canada). The tissue homogenates were centrifuged at 1,600×g for 10 min at 4°C. The supernatant was then used for analysis. Lipid peroxidation in supernatants was estimated by thiobarbituric acid reactive substances (TBARs) assay using a commercially available kit (Abnova, Taipei City, Taiwan) as per the manufacturer’s instructions. Reaction of malondialdehyde (MDA) and other carbonyls with thiobarbituric acid (TBA) under acidic conditions generates chromophores with absorbance at 530 nm.

### Statistical analysis

All data presented are mean ± SEM of 5–7 animals from each treatment group, except for the control group in phase 2 where 3 animals were used, as indicated in the figure legends. MCh curves were fitted using nonlinear regression, and E_max_ values were compared. Statistical analysis was done using GraphPad Prism software (version 4.0). For analyses of blood pressure data, we used a two-way analysis of variance (ANOVA) with Bonferroni’s post-hoc test. For all other studies, a t-test was used for comparison between 2 groups while a one-way ANOVA (with Dunnett’s post-hoc test) was performed for comparison involving 3 or more groups. A p value<0.05 was considered statistically significant.

## Results

### Fried whole egg hydrolysate (FWE-H) reduces blood pressure in SHRs

**Figure 1:**
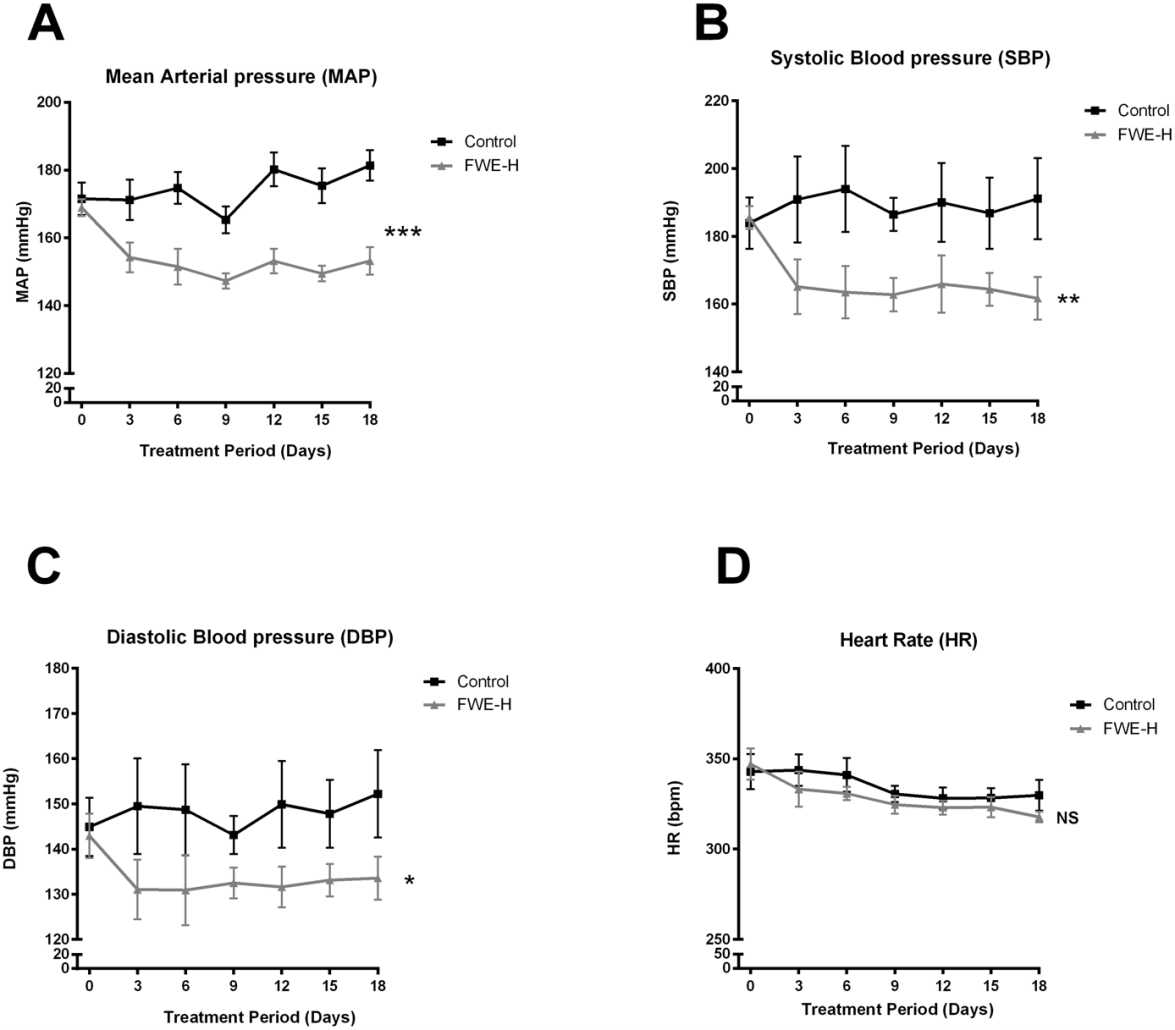
Fried whole egg hydrolysate (FWE-H) reduces BP in SHRs. (A, B, and C) MAP, SBP, and DBP (mmHg) values for control or FWE-H treated (1000 mg/Kg BW) SHRs over a period of 18 days. BP values for each represent the mean BP recorded over a 24 hr period. (D) HR (bpm) of SHRs in treatment groups over 18 days. Treatment with FWE-H significantly lowered MAP (A), SBP (B), and DBP (C) but not heart rate (D). Data represented as mean ± SEM from n = 6–7 animals per treatment group. * indicates p<0.05, ** indicates p<0.01, and *** indicates p<0.001 compared to control. NS indicates not significant compared to the control.

**Figure 2:**
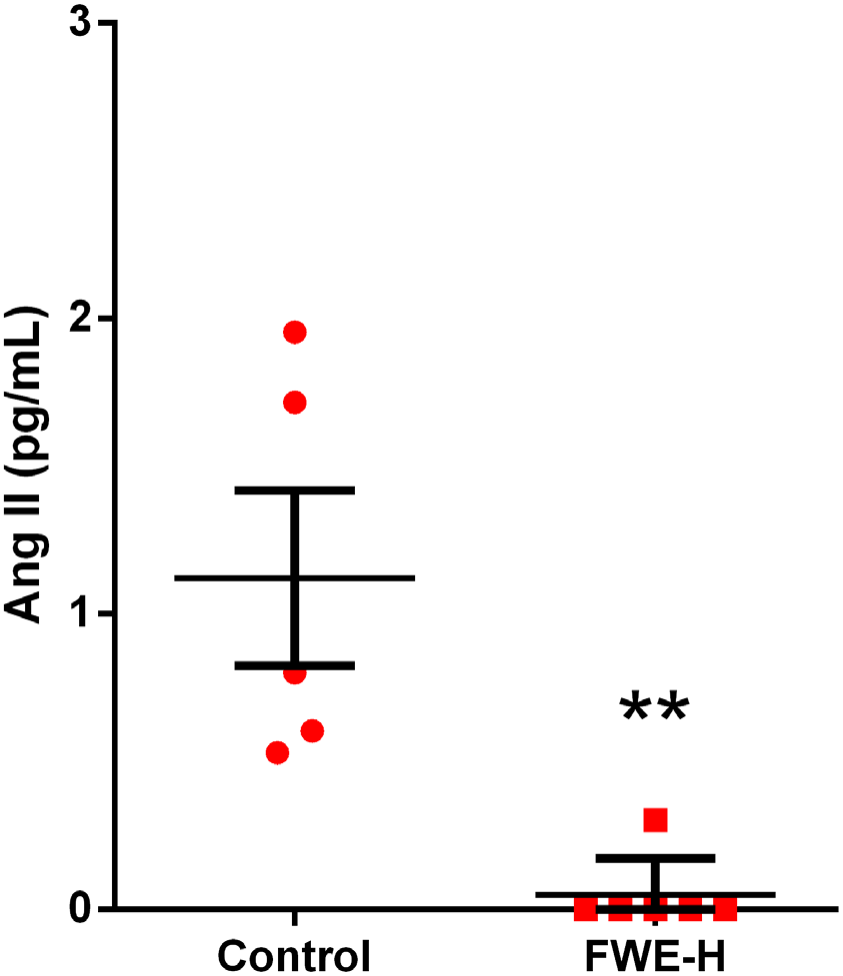
FWE-H attenuates plasma Ang II levels in SHRs. Plasma Ang II levels (pg/mL) from control and FWE-H treated animals are shown. Data represented as mean ± SEM from n = 5–6 animals per treatment group. ** indicates p<0.01 compared to control.

In the beginning of the study (Day 0), the blood pressure was around 170 mmHg in both experimental groups, an indication of established hypertension in these animals. MAP of control animals showed a fluctuating trend reaching 174.2±2.1 mmHg at the end of the study (Day 18), whereas in FWE-H treated group, MAP decreased over time and reached 154.0±2.65 mm Hg, which was significantly lower than the control group (p<0.001, Fig. 1A). A significant reduction (p<0.05) in blood pressure was observed initially on day 6 and continued up to the end of the study period (day 18) with an increased level of significance (p<0.001) observed from day 12 onwards. The changes to MAP involved reductions at the level of both SBP and DBP which were also significantly lower in FWE-H treated animals compared to the control group (Fig. 1B and C). FWE-H did not alter HR compared to the control group (Fig. 1D). The blood pressure lowering effects of FWE-H in SHRs was associated with concomitant changes in circulating Ang II levels. FWE-H treatment reduced plasma Ang II levels significantly (p<0.01) from 1.12±0.30 pg/mL in the control group to 0.05±0.05 pg/mL (Fig. 2). This suggests potential ACE inhibitory properties of FWE-H contributing towards its antihypertensive effects.

### FWE-H induces NO dependent vasorelaxation

**Figure 3:**
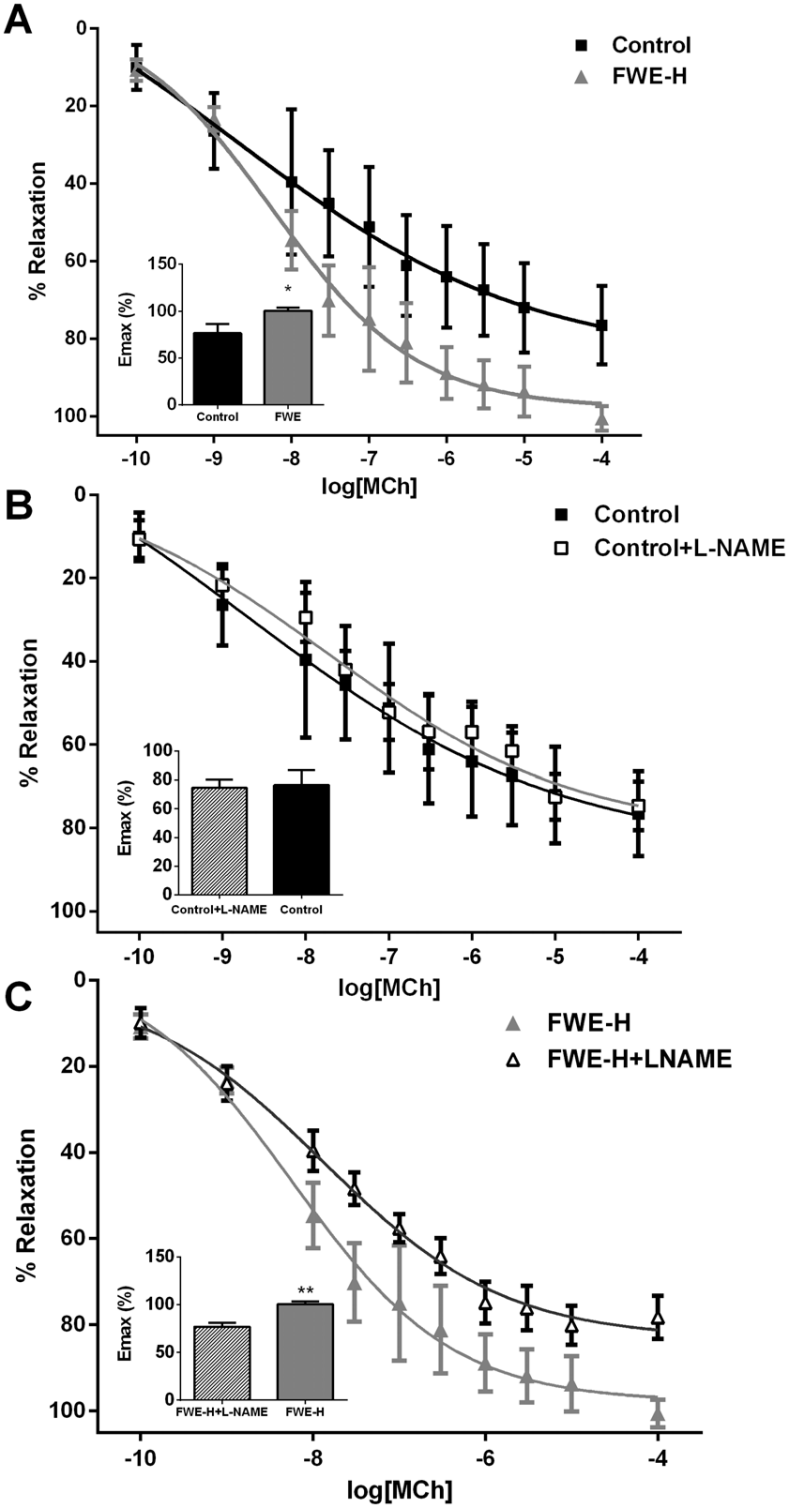
FWE-H treatment induces nitric oxide contribution to vasodilation in mesenteric arteries of SHRs. (A) FWE-H significantly increased maximal vasorelaxation to MCh. Pre-incubation with L-NAME did not change vasorelaxation in control animals (B) while it significantly attenuated vasorelaxation in FWE-H treated animals (C). Data represented as mean ± SEM from n = 4–6 animals per treatment group. * indicates p<0.05, and ** indicates p<0.01 compared to control.

The vascular relaxation to MCh was significantly enhanced by treatment with FWE-H compared to the control group (p<0.05, Fig. 3A). L-NAME, a nitric oxide synthase (NOS) inhibitor, was used to evaluate NO dependent relaxation of mesenteric arteries to MCh. Incubation of mesenteric arteries with L-NAME did not change vasorelaxation in control animals (Fig. 3B), but relaxation was significantly decreased in the treated group (p<0.01, Fig. 3C), suggesting the restoration of NO dependent vasorelaxation in FWE-H group.

### FWE-H attenuates tissue oxidative stress

**Figure 4:**
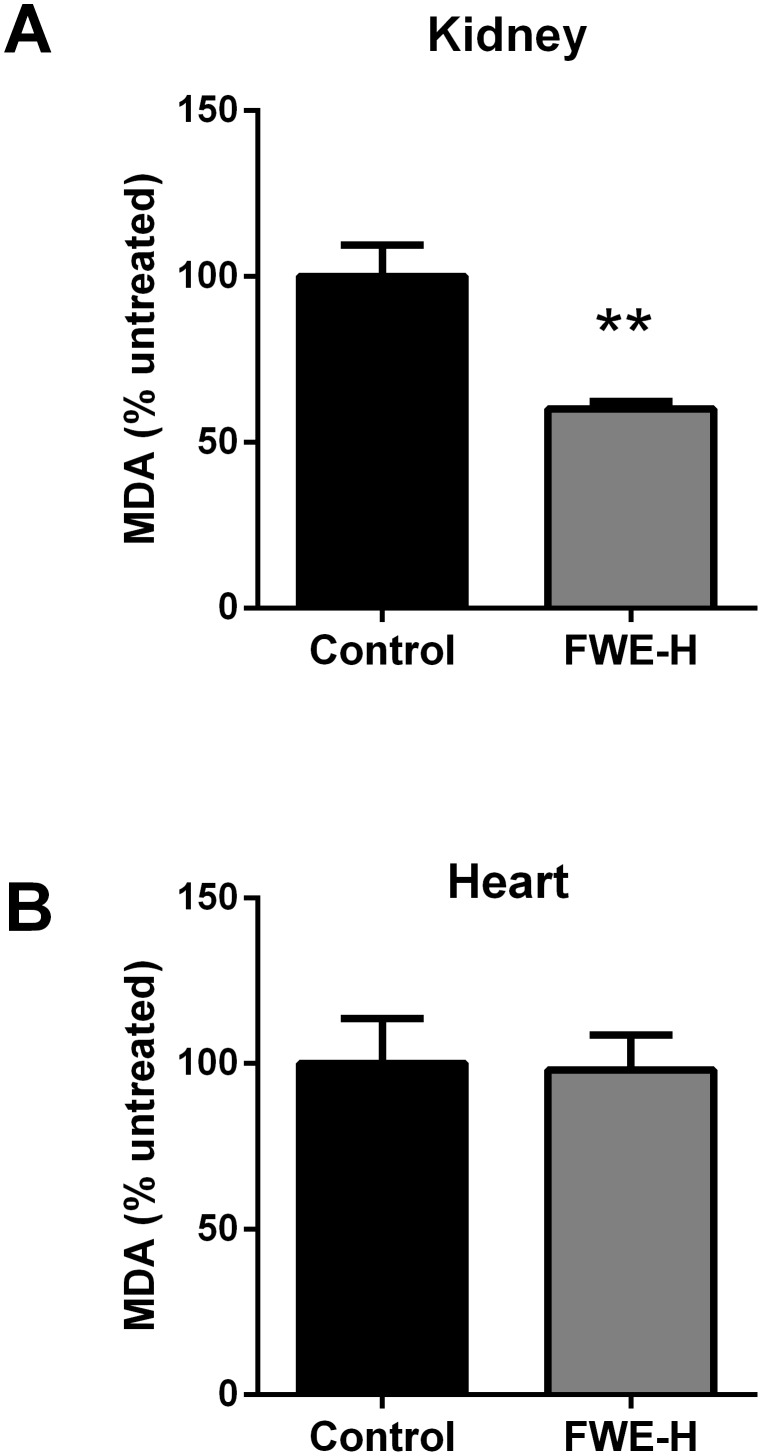
Effects of FWE-H on tissue MDA levels in SHRs. Levels of MDA in kidneys (A) and hearts (B) were estimated and expressed as percentage (%) of control animals. Data represented as mean ± SEM from n = 5–6 animals per treatment group. ** indicates p<0.01 compared to control.

Tissue oxidative stress was evaluated by measuring MDA concentration. In comparison to the control, MDA levels in the kidney (p<0.01; Fig. 4A), but not in the heart (Fig. 4B), were significant reduced.

### FWE-H attenuates plasma TG without affecting cholesterol levels

**Figure 5:**
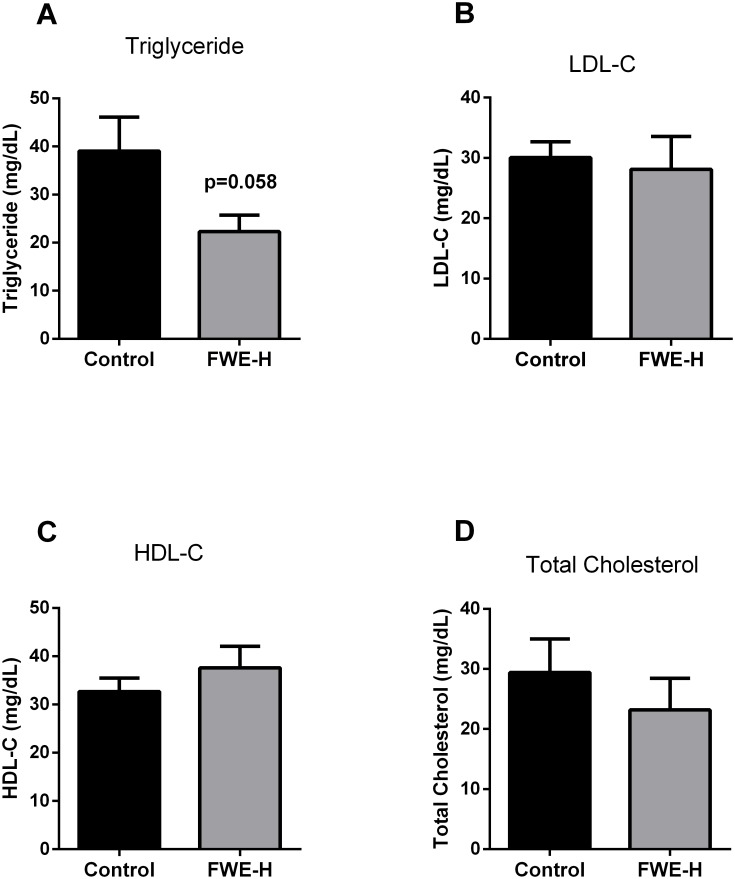
Effects of FWE-H on plasma lipid profile in SHRs. Plasma from control and FWE-H treated animals were analyzed for triglyceride (A), LDL-C (B), HDL-C (C), and total cholesterol (D). Data represented as mean ± SEM from n = 6 animals per group.

FWE-H treated SHRs demonstrated a trend towards lower TG levels compared to the control group (22.32±3.39 mg/dL vs. 39.07±7.05 mg/dL; Fig. 5A); however, plasma cholesterol levels (LDL-C, HDL-C and total C) were not changed (Fig. 5B–D), suggesting no adverse effects of FWE-H on cholesterol levels.

### Non-hydrolyzed fried whole egg did not exert anti-hypertensive effects

**Figure 6:**
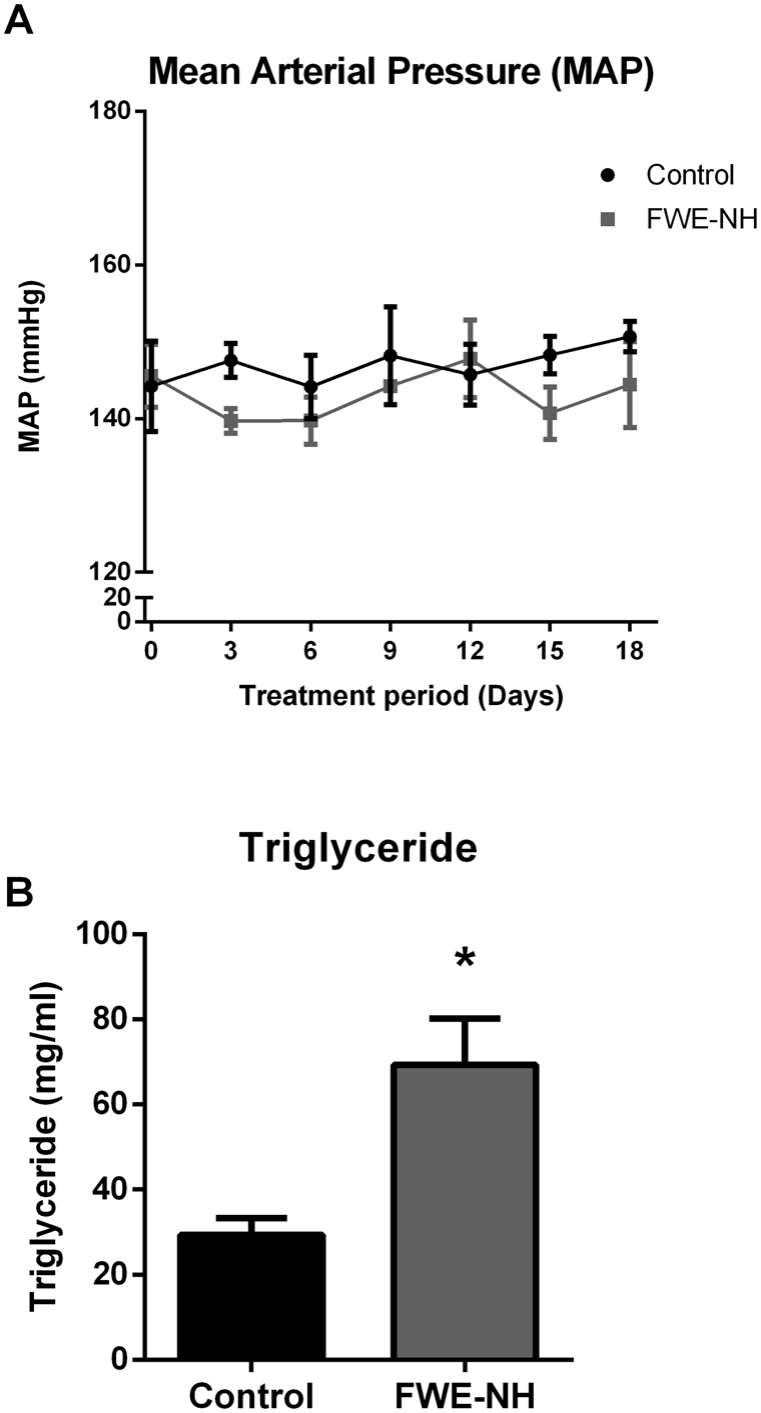
Effects of FWE-NH on BP and plasma triglyceride levels in SHRs. (A) FWE-NH had no effect on MAP compared to the control animals. (B) FWE-NH significantly increased the plasma triglyceride levels in SHRs. Data represented as mean ± SEM from n = 3–6 animals per treatment group. * indicates p<0.05 compared to control.

MAP was not changed in animals fed on FWE-NH (Fig. 6A), indicating that potential antihypertensive compounds may have been formed during *in vitro* enzymatic digestion. Cholesterol levels were also not affected while that of TG was significantly increased (Fig. 6B).

### Hydrolyzed fried egg fractions differentially regulate blood pressure

**Figure 7:**
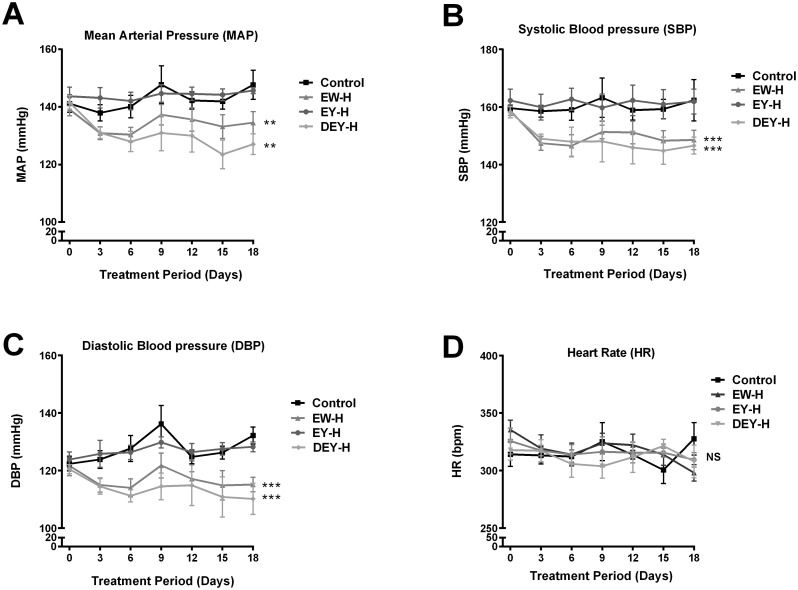
Effects of egg white hydrolysate, egg yolk hydrolysate and defatted egg yolk hydrolysate on BP in SHRs. EW-H, and DEY-H significantly lowered MAP (A), SBP (B), and DBP (C) while EY-H had no effects. None of the treatments altered heart rate (D). Data represented as mean ± SEM from n = 5–7 animals per treatment group. ** indicates p<0.01, and *** indicates p<0.001 compared to control.

**Figure 8:**
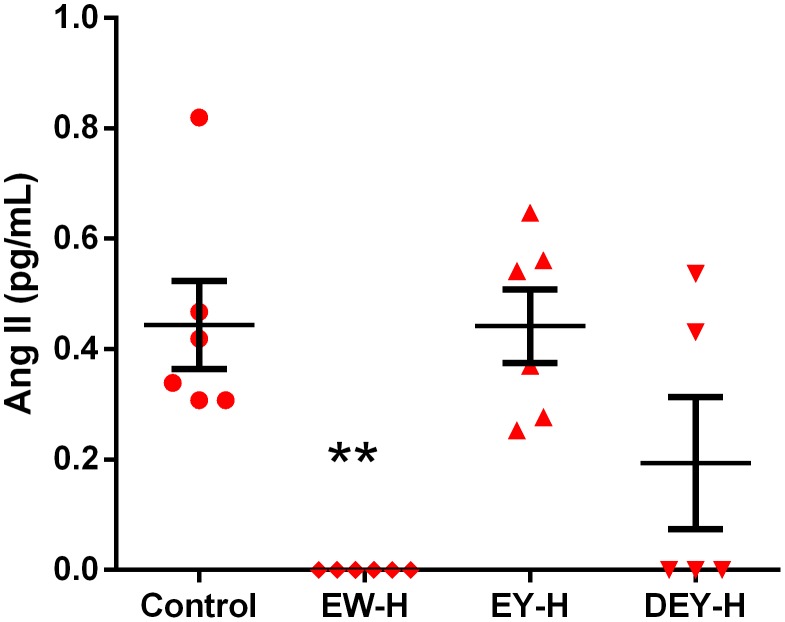
Effects of egg white hydrolysate, egg yolk hydrolysate and defatted egg yolk hydrolysate on plasma Ang II levels in SHRs. Plasma Ang II levels (pg/mL) from control and treated animals are shown. Data represented as mean ± SEM from n = 5–6 animals per treatment group. ** indicates p<0.01 compared to control.

Since FWE-H treatment reduced MAP by ∼30 mmHg in SHRs compared to the control, we next examined the effects of different egg fractions (egg white, egg yolk and defatted egg yolk) on blood pressure to elucidate their individual antihypertensive properties. While MAP of control animals increased by 6 mmHg by the end of the study (day 18), both hydrolyzed fried egg white (EW-H) and hydrolyzed defatted egg yolk (DEY-H) treatments significantly reduced MAP in SHRs compared to the control animals; however, MAP was not affected in egg yolk digest (non-defatted, Fig. 7A). SBP and DBP of these animals followed the same trend as that of MAP (Fig. 7B and C), while heart rate was not affected (Fig. 7D). Consistent with the blood pressure data, plasma Ang II levels were reduced in EW-H and DEY-H treated animals compared to the control and EY-H groups. In fact, plasma Ang II was below the limits of detection in all of the EW-H treated and 3 out of 5 of the DEY-H treated animals (Fig. 8).

### Hydrolyzed fried egg fractions have minimal effects on ex vivo vascular function

Relaxation to MCh was unchanged between groups: E_max_ values for the control, EW-H, DEY-H and EY-H groups were 74.40±13.1, 80.74±7.67, 86.58±4.75, and 91.59±3.28, respectively.

### Effect of fried egg fractions on tissue oxidative stress and plasma lipid profiles

**Figure 9:**
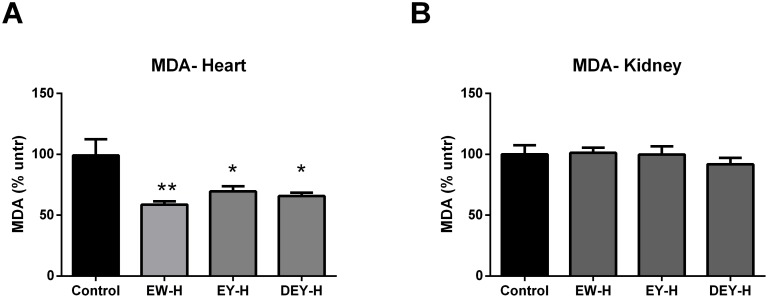
Effects of egg white hydrolysate, egg yolk hydrolysate and defatted egg yolk hydrolysate on tissue MDA levels in SHRs. (A and B) Treatment with all of the egg component hydrolysates significantly reduced MDA levels in hearts (A) without affecting those in the kidneys (B). Data represented as mean ± SEM from n = 4 animals per treatment group. * indicates p<0.05, and ** indicates p<0.01 compared to control.

In contrast to the FWE-H, hydrolyzed egg fractions significantly reduced MDA levels in the hearts of SHRs (Fig. 9A) but not in the kidney (Fig. 9B).

**Figure 10:**
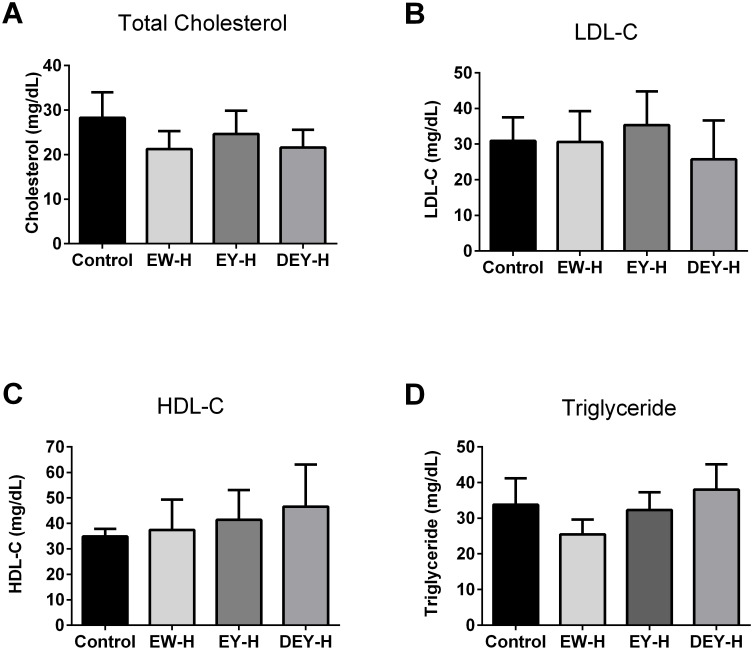
Effects of egg white hydrolysate, egg yolk hydrolysate and defatted egg yolk hydrolysate on plasma lipid profile in SHRs. Plasma from control and treated animals were analyzed for total cholesterol (A), LDL-C (B), HDL-C (C), and triglyceride (D). None of the treatments altered lipid parameters significantly compared to the control. Data represented as mean ± SEM from n = 6 animals per treatment group.

Plasma lipid profiles were not significantly altered in SHRs (Fig. 10A–D), although there was a trend towards reducing total plasma cholesterol and TG in EW-H treated group.

## Discussion

The present study revealed that: (i) administration of fried whole egg hydrolysate (FWE-H), egg white hydrolysate and defatted egg yolk, but not egg yolk hydrolysate (non-defatted) could reduce blood pressure and plasma Ang II levels in SHRs while non-hydrolyzed fried whole egg (FWE-NH) did not change blood pressure suggesting a key role of prior digestion; (ii) fried whole egg hydrolysate (FWE-H) increased NO-dependent vasorelaxation; (iii) Plasma TG levels were reduced by FWE-H, increased by FWE-NH while unaffected by the hydrolyzed fractions; (iv) only the hydrolyzed preparations could attenuate tissue oxidative stress.

SHRs are one of the most widely used animal models of hypertension with pathophysiological similarities to human essential hypertension [13]. Compared to normal rats, SHRs show higher circulating levels of Ang II, impaired vasorelaxation as well as increases in oxidative stress and inflammation [14]. In agreement with our previous work on a modest hypotensive effect in a 3-day study [10], we further confirmed the antihypertensive activity of FWE-H over a longer period of study. Reductions in MAP, SBP, and DBP of SHRs upon oral administration of FWE-H were concomitant with the reduction in plasma Ang II levels at the end of the study. Ang II as a vasoconstrictor is a key contributor to BP regulation and plays a major role in the pathophysiology of hypertension and its associated vascular damage [15].

FWE-H also improved vascular relaxation. Mesenteric arteries as determinants of blood pressure contribute significantly to the alteration of systemic vascular resistance [16],[17]. Therefore, this vascular bed was studied to investigate the effects of these treatments on the alteration of *ex vivo* vascular function. Administration of FWE-H enhanced MCh-induced vasorelaxation compared to the control group which likely involved direct modulation of vascular tone in an endothelial-dependent manner. Multiple factors such as nitric oxide (NO), prostacyclin, and endothelium-derived hyperpolarizing factor (EDHF) contribute to endothelium-dependent relaxation [18]–[20]. L-NAME as an NOS inhibitor was used to further investigate NO-dependent relaxation of mesenteric arteries to MCh. Incubation of mesenteric arteries with L-NAME did not change vasorelaxation in control animals while it was significantly reduced in the FWE-H treated group; suggesting that the enhanced vasorelaxation in FWE-H group was NO-dependent. Whether this increase in NO bioavailability was due to scavenging of reactive oxygen species or increased NO production remains to be identified.

Oxidative stress and its resultant damage contribute to the pathology of cardiovascular disorders including hypertension [21]–[23]. Ang II-induced increases in oxidative stress and endothelin (a potent vasoconstrictor) have been implicated in the onset and development of hypertension [24], while antioxidant treatments have been reported to lower blood pressure in animal models [25],[26]. Lipid peroxidation occurs under conditions of oxidative stress and directly damages cell membranes and generates a number of secondary products including aldehydes, such as MDA, which is taken as a biomarker of oxidative tissue injury [27]. FWE-H reduced MDA levels in kidneys of SHRs which may contribute towards the observed hypotensive effects given the critical role of the kidneys in regulating blood pressure.

In contrast to fried whole egg hydrolysate (FWE-H), non-hydrolyzed fried whole egg (FWE-NH) did not exert anti-hypertensive effects in SHRs, indicating that the generation of bioactive peptides and possibly free amino acids during hydrolysis may be critical for the observed antihypertensive effects. The generation of antihypertensive peptides from egg proteins has been extensively reported in the literature [28]–[30]. In the context of hypertension, numerous ACE-inhibitory [31]–[34], and antioxidant peptides [28],[35],[36] have been identified from different egg protein hydrolysates. Moreover, hypotensive properties of egg protein hydrolysates have also been confirmed in animal studies using SHRs [10],[37]. Following the hydrolysis of FWE, diverse bioactive peptides are generated which may contribute to the observed beneficial effects.

Although non-hydrolyzed fried whole egg also underwent digestion in the digestive system of the animal, this process appears to be inefficient compared to the simulated gastro-intestinal hydrolysis performed *in vitro*. These two hydrolytic processes indeed differ from each other regarding the enzymes present as well as the conditions and duration of the process. The commercially available enzymes used for the *in-vitro* digestion were porcine origin which may generate a different array of peptides compared to the native enzymes present in the rat gastro-intestinal tract. Moreover, *in vitro* digestion has been performed under consistent stirring, which enhances sufficient substrate and enzyme contact and therefore facilitates digestion. The duration of *in vitro* digestion was 3 hours for each enzyme, whereas the gastro-intestinal digestion time in rats is normally between 2 to 4 hours in total [38]. All these factors indicate the key role for prior enzymatic digestion in order to exert maximal beneficial effects *in vivo* from fried whole egg.

We examined the contribution of different fractions of fried egg on blood pressure. Egg white-hydrolysate (EW-H) and defatted egg yolk-hydrolysate (DEY-H), but not egg yolk-hydrolysate (non-defatted, EY-H) significantly reduced MAP, SBP and DBP compared to the control group. This is in accordance with the reported blood lowering effects of an egg white hydrolysate by Miguel *et al.* [39]. Since egg white contains several proteins that are potential sources for bioactive peptides, the beneficial effects may only be observed following liberation of such peptides after hydrolysis. For instance, Miguel and co-workers reported hypotensive properties of ovalbumin derived peptides in SHRs lowering systolic blood pressure in a dose-dependent manner [37]. Another study by Matoba *et al* has also demonstrated similar effects of an egg-derived peptide (RADHPF, obtained upon chymotrypsin mediated digestion of ovalbumin) in inducing NO dependent vasorelaxation in SHRs [40]. In a similar vein, antihypertensive (as well as antioxidant and anti-inflammatory) properties of IRW, a tripeptide derived from hydrolysate of ovotransferrin (an egg white protein), have been established by our group involving biochemical, cell culture and animal studies [12],[41],[42].

Egg yolk proteins have also been validated as anti-hypertensives as shown by the blood pressure lowering effects of oligopeptides from chicken egg yolk hydrolysate [43]. In our study, only treatment with DEY-H but not EY-H reduced blood pressure, indicating potential negation of hypotensive effects by egg yolk lipids. This was intriguing as oleic acid (OA); a major component of neutral lipids in egg yolk (47%), has been shown to exert beneficial cardiovascular effects in some studies. Beneficial effects of OA rich foods on lipid profiles, vascular reactivity and cardiac functions have been reported in animal models [44],[45] as well as several clinical trials [46],[47]. However, these studies generally used oleic acid from vegetable sources such as olive or sunflower and the doses used were usually much higher compared to the dose used in this study [48]. Moreover, oleic acid from vegetable sources had significant antihypertensive effects while intake of oleic acid from animal sources was associated with a significant increase in SBP, further highlighting the role of oleic acid source on its biological actions [48]. Similarly, phospholipids, another key component of egg yolk, have been shown to benefit the lipid profile largely through inhibition of cholesterol absorption in the gut [49]. However, in our study, no beneficial effects on cholesterol were exerted by egg yolk hydrolysates. This is not surprising as the phospholipids present in egg yolk are associated with lipoprotein in the form of complex structures such as low density lipoproteins (LDL) and high density lipoproteins (HDL) [50]. These lipid-protein complexes may not be hydrolyzed completely during *in vitro* digestion, preventing the release of phospholipids to exert their hypocholesterolemic actions. In addition, this would also prevent the release of bioactive compounds (anti-hypertensive proteins/peptides) in egg yolk that could potentially reduce blood pressure. Since egg lipids are concentrated in the yolk, it is also likely that rats in the EY-H group received a higher dose of lipids compared to those in the FWE-H group which could have potentially increased their negative effects on blood pressure regulation in the former group. Moreover, the additional presence of egg white derived bioactive peptides might have counteracted the deleterious effects of lipids in the FWE-H treated rats. These factors may explain why the presence of lipids did not hinder the antihypertensive properties of FWE-H in contrast to those of EY-H.

Our study also showed *in vivo* antioxidant properties of egg hydrolysates (EW-H, EY-H and DEY-H) in heart, but not in the kidney. A previous publication from our group had characterized tyrosine and tryptophan as the two major antioxidants in egg yolk [51]. *In vitro* enzymatic digestion could further enhance the antioxidant activity probably due to the release of further amino acids and bioactive peptides [52]. All the hydrolyzed samples tested in the current study demonstrated reductions in tissue oxidative stress. Interestingly, FWE-H reduced oxidative stress in kidney but not in heart, while the reverse was true in case of the egg fractions (EW-H, EY-H, DEY-H). This may be attributed to the availability of specific antioxidant compounds within the different formulations. The concentrations of bioactive compounds are likely to be different in each of the egg hydrolysate preparations tested. Since the DEY-H and EY-H have similar non-lipid constituents, it is not surprising that they have similar antioxidant effects in the heart. As EW-H and EY-H both showed antioxidant effects it is likely that each contains sufficient levels of bioactive components with antioxidant properties. However, such antioxidant effects were lacking in FWE-H, suggesting that the level of active components might be below the required threshold for biological action (i.e. antioxidant effect). In case of antioxidant effects only observed with FWE-H in kidney, it is probable that there is synergistic effect among various bioactive components present in the different fractions which are unable to exert the similar effect when they are treated alone. Indeed, Manso *et al.* did not observe any significant changes in liver and aorta MDA levels upon administration of hydrolyzed egg white proteins at 1 g/kg BW to SHRs, while at 0.5 g/kg BW aorta MDA levels were reduced significantly compared to controls [53]. These findings highlight the importance of effective dosages of egg proteins for exerting beneficial effects in an intact body. The presence or absence of lipids in egg yolk fractions did not affect the antioxidant properties in the present study, since both EY-H and DEY-H reduced tissue oxidative stress suggesting these effects were independent of the lipid content of the samples.

Another interesting outcome of the present study was the observed change in plasma triglyceride levels upon fried whole egg feeding. We found that feeding non-hydrolyzed fried whole egg changed neither the total cholesterol nor the levels of LDL and HDL-cholesterol while it significantly raised the TG levels. These data appear to contradict the recent report on the beneficial effects of egg-enriched diets on lipid profile of rats [54]. In this study, Sprague-Dawley (SD) rats fed an egg-enriched diet had lower plasma triglycerides, total cholesterol, and LDL-cholesterol concentration than those fed a plain cholesterol diet. Several factors might be involved in the observed discrepancies. Firstly, the SD rat is more susceptible to changes in lipid profile upon feeding different diets than is the SHR [55]. Dose and duration of administered diets are other factors which may further affect the outcome. The average daily dose of administered egg yolk/whole egg in the previous study [54] was more than 15 times the dose used in the present study. In contrast to FWE-NH, FWE-H treatments markedly decreased the TG levels by 40% with no changes in cholesterol compared to the control group. Enzymatic hydrolysis of food derived proteins has shown beneficial effects in lipid metabolism. For instance, a fermented milk preparation improved the serum lipid profile and exerted a hypotensive effect on both rats and humans [56]. Thus, it is possible that in the present study, prior hydrolysis had altered the biophysical and/or biochemical properties of FWE such that lipid absorption was altered and the lipid profile had been beneficially affected. As plasma TG level increases have been associated with risk of cardiovascular disease, the apparent lowering of TG by FWE-H may be an additional protective effect. Indeed, long-term administration of egg white hydrolysate has been reported to reduce TG and total cholesterol in SHRs [53]. In the current study, feeding EW-H also reduced total cholesterol and TG levels by more than 20% (compared to the control group) although this reduction was not statistically significant. It is likely that a synergistic effect might exist among bioactive components present in different egg fractions that together mediate the reduction in TG observed after FWE-H treatment; EW-H and WY-H fractions did not elicit such beneficial effects.

Thus, we demonstrated the role of hydrolysates of fried egg and individual fractions in controlling hypertension through multiple mechanisms of action, involving modulation at the levels of the renin-angiotensin system, nitric oxide, and oxidative stress. While our findings are certainly novel, further research is needed to ascertain the roles of egg lipids and the specific antioxidant potential of different egg fractions to achieve a better understanding of underlying mechanisms. The findings from this study may establish the potential of egg in the management of hypertension and its complications.
